# Soluble ST2 Levels Are Associated with Bleeding in Patients with Severe Leptospirosis

**DOI:** 10.1371/journal.pntd.0000453

**Published:** 2009-06-02

**Authors:** Jiri F. P. Wagenaar, M. Hussein Gasem, Marga G. A. Goris, Mariska Leeflang, Rudy A. Hartskeerl, Tom van der Poll, Cornelis van 't Veer, Eric C. M. van Gorp

**Affiliations:** 1 Department of Internal Medicine, Slotervaart Hospital, Amsterdam, The Netherlands; 2 Department of Internal Medicine, Dr. Kariadi Hospital–Diponegoro University, Semarang, Indonesia; 3 Royal Tropical Institute (KIT), KIT Biomedical Research, Amsterdam, The Netherlands; 4 Center for Experimental and Molecular Medicine, Amsterdam, The Netherlands; Mahidol University, Thailand

## Abstract

**Background:**

Severe leptospirosis features bleeding and multi-organ failure, leading to shock and death. Currently it is assumed that both exaggerated inflammation and immune suppression contribute to mortality in sepsis. Indeed, several proinflammatory cytokines are reported to be induced during leptospirosis. Toll-like receptors, which play an important role in the initiation of an innate immune response, are inhibited by negative regulators including the membrane-bound ST2 (mST2) receptor. Soluble ST2 (sST2) has been implicated to inhibit signaling through mST2. The aim of this study was to determine the extent of sST2 and (pro-) inflammatory cytokine release in patients with severe leptospirosis.

**Methodology and Principal Findings:**

In an observational study, 68 consecutive cases of severe leptospirosis were included. Soluble ST2 and cytokines (TNF-α, IL-1β, IL-6, IL-8, and IL-10) were repeatedly measured. To determine whether blood cells are a source of sST2 during infection, we undertook an *in vitro* experiment: human whole blood and peripheral blood mononuclear cells (PBMC) were stimulated with viable pathogenic *Leptospira*. All patients showed elevated sST2, IL-6, IL-8, and IL-10 levels on admission. Admission sST2 levels correlated with IL-6, IL-8, and IL-10. Thirty-four patients (50%) showed clinical bleeding. Soluble ST2 levels were significantly associated with bleeding overall (OR 2.0; 95%CI: 1.2–3.6) and severe bleeding (OR 5.1; 95%CI: 1.1–23.8). This association was unique, since none of the cytokines showed this correlation. Moreover, sST2 was associated with mortality (OR 2.4; 95%CI: 1.0–5.8). When either whole blood or isolated PBMCs were stimulated with *Leptospira in vitro*, no sST2 production could be detected.

**Conclusions:**

Patients with severe leptospirosis demonstrated elevated plasma sST2 levels. Soluble ST2 levels were associated with bleeding and mortality. *In vitro* experiments showed that (white) blood cells are probably not the source. In this regard, sST2 could be an indicative marker for tissue damage in patients suffering from severe leptospirosis.

## Author Summary

Leptospirosis is a bacterial disease that is mainly spread by rodents and other small mammals. Transmission frequently occurs in (sub-) tropical countries, where environmental circumstances are most favourable. Severe leptospirosis can cause bleeding and vital organ dysfunction. An exaggerated immune response is thought to play an important role in the pathophysiology of leptospirosis. Soluble ST2 (sST2) is thought to inhibit negative regulatory pathways of this response. Soluble ST2 is produced by cells that surround, for example, blood vessels, and several of these blood cells play an important part in the host immune response. In an observational study, we measured the extent of sST2 release in patients suffering from severe leptospirosis. We found that patients that died from leptospirosis displayed higher levels of sST2. Moreover, from this study we have seen that sST2 levels were associated with bleeding, whereas other markers of infection were not. In an experiment, we showed that (white) blood cells did not seem to be the source of sST2 production. Damage to blood vessels is likely to cause bleeding in leptospirosis patients, exposing sST2 producing cells like fibroblasts to the blood stream. Hence, we believe that sST2 may be used as a marker for tissue damage in patients suffering from severe leptospirosis.

## Introduction

Leptospirosis is a worldwide occurring zoonosis [1], reported to be fatal in up to 50% of cases [2]. The disease is caused by spirochetes that are spread by the urine of infected animals, for example rats, mice and cattle amongst others. Survival of *Leptospira* is enhanced in a warm and humid environment, where environmental circumstances are most favourable. Hence prevalence is higher in (sub) tropical countries. Severe leptospirosis is featured by bleeding complications and multi-organ failure, which can eventually lead to shock and even death. Necropsy reports confirm widespread haemorrhaging throughout the body, involving most vital organs and tissues [3]. This haemorrhaging could possibly be the result of capillary wall damage.

Several proinflammatory cytokines, such as TNF-α and IL-12p40 are reported to be induced during infection with *Leptospira* [4],[5]. As well, elevated plasma concentrations of TNF-α have been associated with lethal outcome amongst leptospirosis patients [6], In a hamster model, late expression of the anti-inflammatory cytokines IL-4, TGF-β and IL-10 have been shown [7].

Currently it is assumed that both exaggerated inflammation and immune suppression contribute to an adverse outcome in sepsis [8]. ST2, also designated T1, Fit-1 and DER4, is thought to play a significant role in tuning the host inflammatory response. ST2 is a receptor that is present in two main forms, in the soluble secreted form (sST2) [9] and in a membrane-anchored form (ST2L) [10]. Both are encoded from the *ST2* gene regulated by different promoters [11] and are members of the IL-1 receptor family. ST2 gene expression was identified originally in fibroblasts [9],[12]. Expression has also been detected in several other cells, including Th2 cells, mast cells and macrophages [13]–[16].

ST2L has been reported to attenuate downstream IL-1RI and TLR4 signalling by sequestering MyD88 and MAL (MyD88 adaptor-like) [17]. In contrast, previous work has demonstrated that interleukin (IL)-33 is able to activate NF-κB and MAP kinases by signaling through ST2L [18]. IL-33/ST2L signalling in mast cells and Th2 cells results in the production of Th2-associated cytokines, potentially balancing ongoing inflammatory Th1 responses [18].

The functional role of soluble ST2 has not yet been fully elucidated. Elevated concentrations of sST2 have been found in patients with inflammatory disorders associated with abnormal Th2 mediated responses, in for example, autoimmune diseases [19], asthma [20], idiopathic pulmonary fibrosis [21] and in patients with sepsis [22]. Moreover sST2 have also been proposed as a biomarker for heart failure [23] and elevated levels have been seen to be predictive for clinical outcome in acute myocardial infarction [24].

By using a soluble ST2-Immunoglobulin fusion protein, Sweet et al. demonstrated that this molecule was able to bind macrophages through a putative ST2 receptor, the expression of which was enhanced by LPS stimulation [25]. Furthermore this molecule was shown to suppress LPS-induced proinflammatory response (TNF-α, IL-6 and IL-12) in *vitro* and reduced inflammation and mortality in LPS challenged mice [25]. Administration of soluble ST2-Immunoglobulin fusion protein markedly reduced proinflammatory cytokine production and lethality in intestinal ischemia/reperfusion injury in mice [26]. The anti-inflammatory effect exerted by sST2-Fc was dependent on the elevated production of IL-10. Similar results were seen in hepatic ischemia/reperfusion injury [27]. sST2-Fc fusion protein administration also shown to have beneficial effects in a murine model of collagen-induced arthritis [28]. Asthmatic mice administered with sST2-Fc fusion protein or a soluble ST2 expressing vector showed attenuated production of the Th2 cytokines IL-4 and IL-5 [29],[30], whereas ST2L signaling resulted in the opposite. sST2 has therefore been proposed to act as a decoy receptor for IL-33 [31],[32].

To the best of our knowledge, in previous literature plasma levels of sST2 have not been documented in leptospirosis patients. Hence, in the present study we evaluate sST2, cytokine kinetics and their association with clinical events in a series of severe leptospirosis patients.

## Methods

### Ethics statement

The study protocol was approved by the medical ethic committees of both the Dr. Kariadi hospital- University of Diponegoro, Semarang, Indonesia and the Slotervaart Hospital in The Netherlands. Written informed consent was obtained from all included subjects.

### Patients and design

Consecutive cases of severe leptospirosis were included from February 2005 to September 2006 at the Dr. Kariadi hospital- University of Diponegoro, Semarang, Indonesia. Severe leptospirosis was defined as a hospitalized patient with high clinical suspicion of severe leptospirosis a positive LeptoTek Dri-Dot assay (Biomérieux), presenting with at least one of the following symptoms or signs jaundice, renal failure, thrombocytopenia and/or haemorrhaging..Cases were confirmed by further laboratory testing. Blood samples were taken on hospital admission and during follow up. Plasma was worked up immediately and aliquots were stored at −70°C for further analyses. Twenty control (non-leptospirosis patients) samples were collected among healthy volunteers at the department of internal medicine of the Dr. Kariadi hospital- University of Diponegoro, Semarang, Indonesia.

### Measurements and assays

Soluble ST2 was measured by the commercially available ELISA (R&D systems, Minneapolis, MN). Tumor necrosis factor (TNF)-α, IL-1ß, IL-6, IL-8, IL-10, and IL-12p70 were determined using a cytometric beads array multiplex assay (BD Biosciences, San Jose, CA). The detection limits were as follows, TNF-α, IL-10 (2.5 pg/ml); IL-1ß, IL-6, IL-8 (5 pg/ml); IL-12p70 (10 pg/ml); sST2 (15 pg/ml).

Leptospirosis was confirmed by either a positive culture or microscopic agglutination test (MAT). Tests were considered positive for the MAT with a titre of ≥1∶320 on a single sample, seroconversion or a fourfold or higher increase of the titre in paired samples or a titre ≥1∶80 in a single sample from early deceased patients.

### *In vitro* experiments

To determine whether blood cells are an important source of sST2 during infection, we undertook an *in vitro* experiment. We used either human whole blood or peripheral blood mononuclear cells (PBMCs), which were then stimulated with *Leptospira interrogans* serovar Bataviae strain M, as this serovar is commonly found in the region. This was a fresh, low passage isolate obtained from the Leptospirosis Reference Center in Amsterdam, The Netherlands. For the *in vitro* experiments, bacteria were washed 3 times with RPMI 1640 (Gibco) and counted using a Helber Counting Chamber (Hawksley, Lancing, Sussex, UK) under darkfield microscopy and then resuspended at concentrations of 2.5×10^7^ till 2.5×10^5^ bacteria per ml. Shortly before starting the experiment, heparinized blood was sterilely drawn from multiple healthy donors and diluted 1∶1 in RPMI. PBMC were obtained using Lymphoprep™ (Axis-Shield) according to the manufacturer's guidelines. Whole blood (50 µl per well) or PBMC were divided over each well of a 96-well plate before *Leptospira* concentrates were added. The concentration PBMC was equivalent to 50 µl whole blood per well (approximately 0.5×10^9^ monocytes). Plates were incubated for six hours at 37°C, 5% CO_2_. Following incubation the plates were centrifuged and supernatant was collected and stored at −70°C for further testing. All experiments were performed in quadruplicate. For the negative controls RPMI without bacteria was used.

### Statistical analysis

Continuous variables were presented as medians with corresponding interquartile ranges (IQR) and were statistically evaluated using the non-parametric Mann-Whitney U test. Correlation between soluble ST2, clinical characteristics and cytokine levels in patients were determined using the Spearman correlation coefficient (rho). Associations were calculated using a binary logistic regression analysis and were expressed as odds ratios (OR) and corresponding 95% confidence intervals (CI). An OR >1 indicates that the risk of a clinical event is higher with the increase of biomarker plasma levels and an OR of <1 indicates that the risk of a clinical event is lower with the increase of plasma levels. Associations were further analyzed using the receiver-operating-characteristic (ROC) approach by calculating the area under the ROC curve (AUC). The AUC reflects the probability that a patient with higher plasma levels has a higher chance of the event than a patient with lower plasma levels. Log transformation of the original variables was used to improve the goodness of fit of the model. A p-value of <0.05 was considered to indicate statistical significance. All analyses were done using SPSS (version 15.0, Chicago, Illinois).

## Results

### Characteristics of included patients

In total 68 leptospirosis patients were included, of which 49 (72%) were male. The median age (IQR) was 45 (34–55) years old. In total 16 patients (24%) did not survive, with a median (IQR) time to death of 3 days post hospital admission. On average patient's symptoms had started at a median of 7 days pre hospital admission. Clinical manifestations/symptoms included jaundice (75%), thrombocytopenia (platelets <100×10^9^: 65%), oliguria (19%) and anuria (4%). Median leucocyte, platelet, creatinine and bilirubin levels were: 15×10^9^/L, 69×10^9^/L, 412 µmol/L and 113 µmol/L respectively. All patients had MAT serologically confirmed leptospirosis, with the most frequently identified serogroups being Bataviae (19), Icterohaemorrhagiae (18) and Ballum (2).

**Figure 1:**
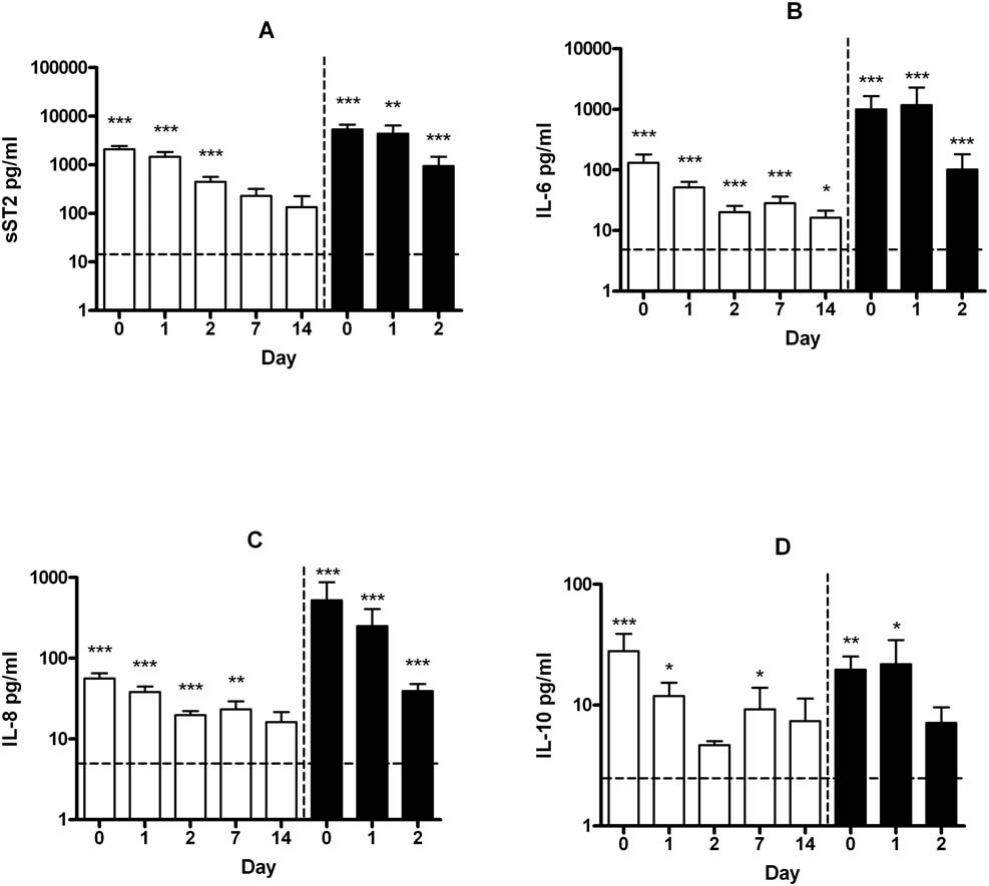
Soluble ST2 and cytokine dynamics in patients with severe leptospirosis. The bar graphs show mean soluble ST2 and cytokine plasma levels for survivors (white) and non-survivors (black). The error bars indicate the standard error of the mean (SEM). The horizontal dotted line represents the detection limit of the assays. Asterisks in the figure indicate the strength of the statistical difference from healthy controls (* p<0.05, ** p<0.001, *** p<0.0001; Mann-Whitney U test).

All patients showed elevated sST2, IL-6, IL-8 and IL-10 levels on admission, presented in Table 1. TNF-α, IL-1β and IL-12p70 levels taken at hospital admission were either very low or undetectable and were not significantly different from the healthy controls (data not shown). Patients that died from leptospirosis (n = 16) had significantly further elevated plasma levels on admission compared to the survivors, sST2 (p = .006), IL-6 (p = .003) and IL-8 (p = .003). However, IL-10 (p = .64) was not found to be significantly elevated. Figure 1 shows the dynamics of sST2, IL-6, IL-8 and IL-10. Circulating sST2 levels in the survivors showed a peak at day 0 after which levels gradually decreased and normalized on day 7. However, patients who died during their hospital stay displayed continuously high plasma levels of sST2 until death occurred. Table 2 shows that admission sST2 levels were significantly correlated with levels of IL-6 (rho 0.45; p = .001), IL-8 (rho 0.72; p<.0001), IL-10 (rho 0.56; p<.0001) and CRP (rho 0.50; p<.0001) also taken at admission. Admission sST2 levels resulted in moderate correlations with respiratory rate (rho −0.25; p = .04), platelets (rho −0.25; p = .04) creatinin (rho 0.33; p = .007), AST (rho 0.46; p = .001) levels and (severe) haemorrhaging (haemorrhaging: rho 0.34; p = .005, severe haemorrhaging: rho 0.32; p = .008).

**Table 1:** Soluble ST2 and cytokines on admission in patient with severe leptospirosis.

Marker (pg/ml)	All (n = 68)	Survivors (n = 52)	Non-survivors (n = 16)	Controls	p-value
	A	B	C	D	BC
**sST2**	1480 (502–4378)	1203 (285–2773)	3596 (1452–8590)	<15	0.006
**IL-6**	45 (17–135)	27 (16–74)	133 (52–430)	<5	0.003
**IL-8**	40 (16–98)	32 (51–182)	81 (51–182)	<5	0.003
**IL-10**	7 (4–18)	6 (4–17)	8 (4–37)	<2.5	0.64

Abbreviations: IQR, interquartile range. Values represent medians with the corresponding IQR range. Statistical difference between survivors and non-survivors was calculated using the Mann-Whitney U test. A p-value <0.05 was considered significant.

**Table 2:** Correlation between soluble ST2 (sST2), clinical markers and cytokines on day of admission.

Variable	Serum sST2	
	rho	p-value
Pulse	0.20	0.1
RR	−0.25	0.04
Leucocytes	−0.05	0.7
Platelets	−0.25	0.04
Creatinin	0.33	0.007
AST	0.46	0.001
ALT	−0.016	0.9
CRP	0.50	<0.0001
Haemorrhaging	0.34	0.005
Mild	0.10	0.4
Severe	0.32	0.008
TNF-α	0.17	0.2
IL-1β	0.14	0.3
IL-12p70	0.04	0.8
IL-6	0.45	0.001
IL-8	0.72	<0.0001
IL-10	0.56	<0.0001

Abbreviations: RR, respiratory rate; CRP, C-reactive protein. The correlation coefficient (rho) is calculated by the non-parametric Spearman's rank correlation test. A p-value<0.05 was considered significant.

### Bleeding is associated with increased sST2 plasma levels

Since bleeding is an important feature of severe leptospirosis we were interested whether this event was also associated with sST2 levels. In total 34 patients (50%) showed signs of bleeding. We found mild haemorrhages (petechiae, ecchymoses and epistaxis) in 23 cases and severe haemorrhages (gastrointestinal, melaena, gum bleeding, hemoptysis and heamaturia) in 10 cases. To determine whether elevated sST2 levels were associated with bleeding, we performed a binary logistic regression analysis and calculated the area under the ROC curve (AUC), see Table 3. Elevated sST2 levels were significantly associated with overall haemorrhaging (mild and severe) (OR 2.0; 95%CI: 1.2–3.6, p = .01; AUC: 0.70, p = .006) and severe haemorrhaging (OR 5.1; 95%CI: 1.1–23.8, p = .04; AUC: 0.76, p = .009), but not with mild bleeding alone (OR 1.3; 95%CI: 0.76–2.2, p = .3; AUC: 0.6, p = .5). As we log-transformed the original variables, this means that for a ten-fold increase of plasma sST2 levels, the odds of developing mild or severe bleeding will be 2.0 times higher, and the odds of developing severe bleeding 5.1 times higher. None of the cytokines were significantly associated with (severe) haemorrhaging (see Table 3).

**Table 3:** Association between soluble ST2 (sST2) bleeding and mortality.

Variable	OR (95%CI)	p-value	AUC	p-value
*Bleeding*				
**10-log sST2**	2.0 (1.2–3.6)	0.01	0.70	0.006
**10-log IL-6**	1.8 (0.9–3.7)	0.1	0.61	0.1
**10-log IL-8**	2.6 (1.0–7.4)	0.06	0.62	0.08
**10-log IL-10**	1.3 (0.5–3.3)	0.6	0.55	0.5
*Severe bleeding*				
**10-log sST2**	5.1 (1.1–24)	0.04	0.76	0.009
**10-log IL-6**	2.0 (0.83–4.9)	0.1	0.66	0.1
**10-log IL-8**	2.4 (0.77–7.7)	0.1	0.65	0.1
**10-log IL-10**	2.0 (0.6–6.6)	0.3	0.61	0.3
*Mortality*				
**10-log sST2**	2.4 (1.0–5.8)	0.05	0.73	0.006
**10-log IL-6**	3.2 (1.4–7.7)	0.008	0.74	0.003
**10-log IL-8**	6.9 (1.8–27)	0.005	0.75	0.003
**10-log IL-10**	1.3 (0.5–3.9)	0.58	0.58	0.4

Abbreviations: OR, odds ratio; CI, confidence interval, AUC, area under the ROC curve (receiver operating characteristic). Associations are presented as OR with 95% confidence interval and AUC values. A p-value<0.05 was considered significant.

### Soluble ST2 and cytokine levels are associated with mortality

The association between plasma levels sST2, cytokines and mortality was calculated using a binary logistic regression (OR, 95% CI) and a ROC approach (AUC). The odds of patients with leptospirosis dying increased by 2.4 (95%CI: 1.0–5.8, p = .05) with a ten-fold increase of plasma sST2 levels with an AUC of 0.73 (p = .006). As well the odds of patients with leptospirosis dying increased by 3.2 (95%CI: 1.4–7.7, p = .008) with an AUC of 0.74 (p = .003), with a ten-fold increase of plasma IL-6 levels. With a ten-fold increase of plasma IL-8 there was an odds of 6.9 (95%CI: 1.8–27, p = .005) and an AUC of 0.75 (p = .003). The anti-inflammatory cytokine IL-10 failed to reach significance (OR 1.3; 95%CI: 0.5–3.9, p = .58; AUC: 0.58, p = .40), see Table 3.

### Soluble ST2 is not released in whole blood after stimulation with viable *Leptospira*

**Figure 2:**
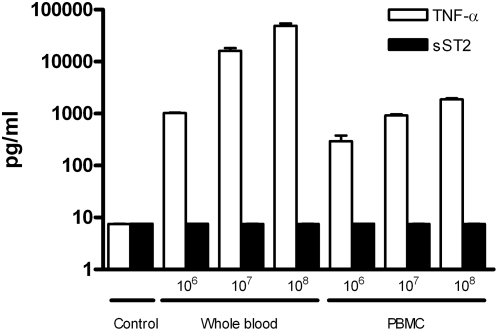
In vitro stimulation of whole blood or peripheral blood mononuclear cells (PBMC) with pathogenic *Leptospira*. This bar graph shows mean TNF-α (white) and sST2 (black) levels with standard error of the mean (SEM) for each group. Either human whole blood or peripheral blood mononuclear cells (PBMC) were incubated with various concentrations pathogenic *Leptospira*. Controls were not incubated with *Leptospira*. Soluble ST2 (sST2) and TNF-α levels were measured in supernatant after 6 hours incubation.

To evaluate whether blood cells are an important source of sST2 during infection, we undertook an *in vitro* experiment. We used a fresh viable pathogenic isolate of *Leptospira interrogans* serovar Bataviae strain M. When either human whole blood or isolated PBMCs were stimulated with different concentrations bacteria, we could not detect sST2 production after 6 hours incubation (Figure 2). The same held true for the negative controls. In contrast, high levels TNF-α were measured as dose dependent upon stimulation with viable *Leptospira*.

## Discussion

This study reports elevated sST2 levels in patients with severe leptospirosis. Soluble ST2 levels correlated with other indicators of inflammation. A unique, significant association between sST2 and bleeding was observed. As well soluble ST2, IL-6 and IL-8 levels were all associated with poor outcome in leptospirosis patients.

Previous work has reported elevated sST2 plasma levels in fifteen septic patients, but in this study no association with mortality was found [22]. Becerra et al. reported elevated sST2 levels in patients suffering from dengue fever, but in the convalescent samples sST2 levels were normalized [33]. Since all patients in this study survived and had only mild disease, no associations with regard to disease severity and outcome could be found. The data presented here extends these earlier studies, with findings of elevated sST2 levels during infection in a larger, homogeneous group of patients.

Leptospirosis patients yielded elevated levels of IL-6 and IL-8 associated with mortality in the present study which were stronger than sST2. From the literature, several studies have found similar results presenting data on the association between cytokine levels and poor outcome in septic patients [34],[35]. However, in these studies the range of cytokine levels from survivors and non-survivors often overlapped, which means that although the cytokines are associated with poor outcomes, they are of little or no prognostic value [36]. In a study by Chierakul et al. elevated TNF-α and IL-12p40 levels were reported in 28 patients with mild leptospirosis [4]. The biologically active IL-12p70 heterodimer was detected in only 4 patients. An other study reported increased TNF-α levels in four out of eighteen leptospirosis patients [6] . In this small study, TNF-α was found to be associated with disease severity and poor outcome. In contrast, in the current work TNF-α, IL-1β and IL-12p70 concentrations were either very low or undetectable and did not differ from the controls. The fact that we could not confirm the association between poor outcome and TNF-α can be explained by the fact that our patients presented in late stage disease and TNF-α is considered to be an early response cytokine.

It is interesting to speculate why (severe) bleeding was found to be associated with sST2 plasma levels. Weinberg et al. identified sST2 release in response to myocardial infarction and suggested that sST2 participates in the cardiovascular response to injury of the cardiomyocytes [37]. Disruption of the endothelial cell barrier, a possible explanation for the haemorrhages found in severe leptospirosis, exposes underlying fibroblasts. In this light, sST2 could be an indicative marker for tissue injury given the fact that serum stimulation of resting fibroblasts results in sST2 release [9]. Our *in vitro* experiments with pathogenic *Leptospira* showed that, at least in the early phase, blood is not the source of sST2 production. These findings were in line with previous findings of our group in which we found that membrane bound ST2 is upregulated on monocytes when whole blood is incubated with LPS, while sST2 remains undetectable in blood plasma after 24 hour whole blood LPS stimulation [38].

In conclusion, in patients with severe leptospirosis we demonstrated elevated plasma sST2 levels that normalized during follow-up and were associated with mortality. Interestingly sST2 was the only marker that was associated with (severe) bleeding. More research is warranted to elucidate the function of sST2 in the innate immune response to *Leptospira* and to evaluate its value as a marker for tissue damage in severely ill patients.

## Supporting Information

Checklist S1 STROBE Checklist
(0.07 MB DOC)

## References

[1] Levett PN (2001). Leptospirosis.. Clin Microbiol Rev.

[2] Segura ER, Ganoza CA, Campos K, Ricaldi JN, Torres S (2005). Clinical spectrum of pulmonary involvement in leptospirosis in a region of endemicity, with quantification of leptospiral burden.. Clin Infect Dis.

[3] Arean VM (1962). The pathologic anatomy and pathogenesis of fatal human leptospirosis (Weil's disease).. Am J Pathol.

[4] Chierakul W, de Fost M, Suputtamongkol Y, Limpaiboon R, Dondorp A (2004). Differential expression of interferon-gamma and interferon-gamma-inducing cytokines in Thai patients with scrub typhus or leptospirosis.. Clin Immunol.

[5] Estavoyer JM, Racadot E, Couetdic G, Leroy J, Grosperrin L (1991). Tumor necrosis factor in patients with leptospirosis.. Rev Infect Dis.

[6] Tajiki H, Salomao R (1996). Association of plasma levels of tumor necrosis factor alpha with severity of disease and mortality among patients with leptospirosis.. Clin Infect Dis.

[7] Vernel-Pauillac F, Merien F (2006). Proinflammatory and immunomodulatory cytokine mRNA time course profiles in hamsters infected with a virulent variant of Leptospira interrogans.. Infect Immun.

[8] van der Poll T, Opal SM (2008). Host-pathogen interactions in sepsis.. Lancet Infect Dis.

[9] Tominaga S (1989). A putative protein of a growth specific cDNA from BALB/c-3T3 cells is highly similar to the extracellular portion of mouse interleukin 1 receptor.. FEBS Lett.

[10] Yanagisawa K, Takagi T, Tsukamoto T, Tetsuka T, Tominaga S (1993). Presence of a novel primary response gene ST2L, encoding a product highly similar to the interleukin 1 receptor type 1.. FEBS Lett.

[11] Iwahana H, Yanagisawa K, Ito-Kosaka A, Kuroiwa K, Tago K (1999). Different promoter usage and multiple transcription initiation sites of the interleukin-1 receptor-related human ST2 gene in UT-7 and TM12 cells.. Eur J Biochem.

[12] Yanagisawa K, Tsukamoto T, Takagi T, Tominaga S (1992). Murine ST2 gene is a member of the primary response gene family induced by growth factors.. FEBS Lett.

[13] Xu D, Chan WL, Leung BP, Huang F, Wheeler R (1998). Selective expression of a stable cell surface molecule on type 2 but not type 1 helper T cells.. J Exp Med.

[14] Moritz DR, Rodewald HR, Gheyselinck J, Klemenz R (1998). The IL-1 receptor-related T1 antigen is expressed on immature and mature mast cells and on fetal blood mast cell progenitors.. J Immunol.

[15] Oshikawa K, Yanagisawa K, Tominaga S, Sugiyama Y (2002). ST2 protein induced by inflammatory stimuli can modulate acute lung inflammation.. Biochem Biophys Res Commun.

[16] Bergers G, Reikerstorfer A, Braselmann S, Graninger P, Busslinger M (1994). Alternative promoter usage of the Fos-responsive gene Fit-1 generates mRNA isoforms coding for either secreted or membrane-bound proteins related to the IL-1 receptor.. EMBO J.

[17] Brint EK, Xu D, Liu H, Dunne A, McKenzie AN (2004). ST2 is an inhibitor of interleukin 1 receptor and Toll-like receptor 4 signaling and maintains endotoxin tolerance.. Nat Immunol.

[18] Schmitz J, Owyang A, Oldham E, Song Y, Murphy E (2005). IL-33, an interleukin-1-like cytokine that signals via the IL-1 receptor-related protein ST2 and induces T helper type 2-associated cytokines.. Immunity.

[19] Kuroiwa K, Arai T, Okazaki H, Minota S, Tominaga S (2001). Identification of human ST2 protein in the sera of patients with autoimmune diseases.. Biochem Biophys Res Commun.

[20] Oshikawa K, Kuroiwa K, Tago K, Iwahana H, Yanagisawa K (2001). Elevated soluble ST2 protein levels in sera of patients with asthma with an acute exacerbation.. Am J Respir Crit Care Med.

[21] Tajima S, Oshikawa K, Tominaga S, Sugiyama Y (2003). The increase in serum soluble ST2 protein upon acute exacerbation of idiopathic pulmonary fibrosis.. Chest.

[22] Brunner M, Krenn C, Roth G, Moser B, Dworschak M (2004). Increased levels of soluble ST2 protein and IgG1 production in patients with sepsis and trauma.. Intensive Care Med.

[23] Weinberg EO, Shimpo M, Hurwitz S, Tominaga S, Rouleau JL (2003). Identification of serum soluble ST2 receptor as a novel heart failure biomarker.. Circulation.

[24] Shimpo M, Morrow DA, Weinberg EO, Sabatine MS, Murphy SA (2004). Serum levels of the interleukin-1 receptor family member ST2 predict mortality and clinical outcome in acute myocardial infarction.. Circulation.

[25] Sweet MJ, Leung BP, Kang D, Sogaard M, Schulz K (2001). A novel pathway regulating lipopolysaccharide-induced shock by ST2/T1 via inhibition of Toll-like receptor 4 expression.. J Immunol.

[26] Fagundes CT, Amaral FA, Souza AL, Vieira AT, Xu D (2007). ST2, an IL-1R family member, attenuates inflammation and lethality after intestinal ischemia and reperfusion.. J Leukoc Biol.

[27] Yin H, Huang BJ, Yang H, Huang YF, Xiong P (2006). Pretreatment with soluble ST2 reduces warm hepatic ischemia/reperfusion injury.. Biochem Biophys Res Commun.

[28] Leung BP, Xu D, Culshaw S, McInnes IB, Liew FY (2004). A novel therapy of murine collagen-induced arthritis with soluble T1/ST2.. J Immunol.

[29] Lohning M, Stroehmann A, Coyle AJ, Grogan JL, Lin S (1998). T1/ST2 is preferentially expressed on murine Th2 cells, independent of interleukin 4, interleukin 5, and interleukin 10, and important for Th2 effector function.. Proc Natl Acad Sci U S A.

[30] Oshikawa K, Yanagisawa K, Tominaga S, Sugiyama Y (2002). Expression and function of the ST2 gene in a murine model of allergic airway inflammation.. Clin Exp Allergy.

[31] Hayakawa H, Hayakawa M, Kume A, Tominaga S (2007). Soluble ST2 blocks interleukin-33 signaling in allergic airway inflammation.. J Biol Chem.

[32] Sanada S, Hakuno D, Higgins LJ, Schreiter ER, McKenzie AN (2007). IL-33 and ST2 comprise a critical biomechanically induced and cardioprotective signaling system.. J Clin Invest.

[33] Becerra A, Warke RV, de BN, Rothman AL, Bosch I (2008). Elevated levels of soluble ST2 protein in dengue virus infected patients.. Cytokine.

[34] Calandra T, Gerain J, Heumann D, Baumgartner JD, Glauser MP (1991). High circulating levels of interleukin-6 in patients with septic shock: evolution during sepsis, prognostic value, and interplay with other cytokines. The Swiss-Dutch J5 Immunoglobulin Study Group.. Am J Med.

[35] Pinsky MR, Vincent JL, Deviere J, Alegre M, Kahn RJ (1993). Serum cytokine levels in human septic shock. Relation to multiple-system organ failure and mortality.. Chest.

[36] Angus DC, Wax RS (2001). Epidemiology of sepsis: an update.. Crit Care Med.

[37] Weinberg EO, Shimpo M, De Keulenaer GW, MacGillivray C, Tominaga S (2002). Expression and regulation of ST2, an interleukin-1 receptor family member, in cardiomyocytes and myocardial infarction.. Circulation.

[38] van 't Veer C, van den Pangaart PS, van Zoelen MA, de KM, Birjmohun RS (2007). Induction of IRAK-M is associated with lipopolysaccharide tolerance in a human endotoxemia model.. J Immunol.

